# Combined chemo-endocrine therapy as a potential new option for HR+/HER2− advanced breast cancer: a prospective study of fulvestrant plus oral vinorelbine

**DOI:** 10.20892/j.issn.2095-3941.2022.0702

**Published:** 2023-05-04

**Authors:** Xue Wang, Jian Yue, Yikun Kang, Zhong Dai, Jie Ju, Jiayu Wang, Pin Zhang, Fei Ma, Binghe Xu, Peng Yuan

**Affiliations:** 1 Department of VIP Medical Services, National Cancer Center/National Clinical Research Center for Cancer/Cancer Hospital, Chinese Academy of Medical Sciences and Peking Union Medical College, Beijing 100021, China; 2 Department of Medical Oncology, National Cancer Center/National Clinical Research Center for Cancer/Cancer Hospital, Chinese Academy of Medical Sciences and Peking Union Medical College, Beijing 100021, China; 3 Department of Comprehensive Oncology, Huanxing Cancer Hospital, Beijing 100026, China; 4 Department of Medical Oncology and Clinical Trial Center, National Cancer Center/National Clinical Research Center for Cancer/Cancer Hospital, Chinese Academy of Medical Sciences and Peking Union Medical College, Beijing 100021, China

**Keywords:** HR+/HER2− breast cancer, recurrence, metastasis, fulvestrant, oral vinorelbine

## Abstract

**Objective:**

Endocrine therapy with fulvestrant has shown synergistic antitumor effects with some chemotherapy drugs *in vitro*. This study evaluated the efficacy and safety of fulvestrant with vinorelbine in patients with hormone receptor positive (HR+)/human epidermal growth factor receptor-2-negative (HER2−) recurrent or metastatic breast cancer.

**Methods:**

Patients were intramuscularly administered fulvestrant 500 mg (day 1 per cycle for 28 days) and oral vinorelbine (60 mg/m^2^ on days 1, 8, and 15 of each cycle). The primary endpoint was progression-free survival (PFS). Secondary endpoints included overall survival, objective response rate, disease control rate, duration of response, and safety.

**Results:**

A total of 38 patients with HR+/HER2− advanced breast cancer included in the study were followed up for a median time of 25.1 months. The overall median PFS was 9.86 months [95% confidence interval (CI) 7.2–23.13], and the median PFS of the first-line and the second-line treatment population was 20.73 months (95% CI 9.82 to NR) and 4.27 months (95% CI 3.68 to NR), respectively. Most adverse events reported were of grade 1/2, and none were of grade 4/5.

**Conclusions:**

This is the first exploratory study of a fulvestrant and oral vinorelbine regimen in the treatment of HR+/HER2− recurrent and metastatic breast cancer. The combination chemo-endocrine therapy was efficacious, safe, and promising for patients with HR+/HER2− advanced breast cancer.

## Introduction

Endocrine therapy has been reported to elicit prognostic improvement in hormone receptor positive (HR+)/human epidermal growth factor receptor-2-negative (HER2−) advanced breast cancer. Single drug endocrine therapy is associated with a median progression free survival (PFS) of 16.6 months^1^. Moreover, the combination of endocrine therapy with cyclin-dependent kinase (CDK)4/6 inhibitors (CDK4/6i) prolongs PFS and overall survival^2^. However, a large proportion of patients are susceptible to drug withdrawal, owing to severe hematological toxicity and poor tolerability with CDK4/6i^3^. In addition, some CDK4/6i drugs are not readily available in China, and hence most patients in China with HR+/HER2− advanced breast cancer receive single endocrine therapy.

However, the challenge of endocrine drug resistance persists in clinical practice, primarily for 2 reasons. First, heterogeneity exists among tumors: HR+ breast cancer tissues may contain hormone receptor negative (HR−) tumor cells that are not responsive to endocrine therapy^4^. Second, HR+ tumor cells themselves may have primary or secondary endocrine drug resistance. The mechanism of drug resistance is complicated and may be associated with the activation of multiple signaling pathways, which remains under exploration^5^. Fulvestrant, an endocrine agent, has been observed to have synergistic effects with a variety of chemotherapeutic drugs^6^. In addition, chemotherapy affects HR− tumor cells, thus enhancing the effects of endocrine therapy and delaying the occurrence of endocrine resistance of HR+/HER2− tumor cells.

Because traditional chemotherapeutic drugs are administered intravenously and require hospitalization, they may cause severe adverse events (AEs). Oral chemotherapeutic drugs are currently preferred because of their convenience and low toxicity. Therefore, we performed a prospective study of fulvestrant in combination with oral vinorelbine to evaluate the efficacy and safety of this combination in patients with HR+/HER2− advanced breast cancer, and to find whether this novel dual-therapy might further improve the prognosis of patients with advanced breast cancer receiving routine endocrine therapy.

## Materials and methods

### Study design and patients

This was a prospective, single-center, single-arm exploratory clinical study initiated by the principal investigators in the Cancer Hospital Chinese Academy of Medical Sciences. The main inclusion criteria were patients (1) 18–70 years of age, with Eastern Cooperative Oncology Group scores of 0–2; (2) who were diagnosed histologically or cytologically with locally recurrent or metastatic breast cancer; (3) who had recurrence after adjuvant endocrine therapy for more than 1 year or progression after first-line endocrine or chemotherapy; (4) who were not candidates for radical surgery or radiotherapy, and had expected survival time longer than 3 months; (5) who were estrogen receptor positive (ER+) and/or progesterone receptor positive (PR+) (ER ≥ 1% and/or PR ≥ 1%), and HER2 negative (HER2− or low expression of HER2 with no fluorescence in situ hybridization amplification); (6) who had at least 1 measurable lesion, in accordance with the response evaluation criteria in solid tumors (RECIST) version 1.1, as identified through an imaging examination within 2 weeks before enrollment; and (7) who had adequate organ function. The main exclusion criteria were patients with (1) brain metastasis with uncontrollable symptoms; (2) oral drug absorption disorder; (3) rapid organ invasion (such as lesions in the lung and/or liver over more than half the organ area, or liver dysfunction, etc.); and (4) any other malignant tumor diagnosed within 3 years. Because this was an exploratory phase II investigator-initiated-trial, the sample size was not calculated, and 30 patients were planned to be enrolled in the study protocol.

This study was approved by the Ethics Committee of the Cancer Hospital Chinese Academy of Medical Sciences (CAMS) on January 18, 2018 and was registered with the clinical trial registry (ClinicalTrials.gov identifier: NCT 03939871). After approval by CAMS, the study prospectively enrolled 38 patients. Written signed informed consent was obtained from patients before enrollment in the study. The trial was conducted in compliance with the Declaration of Helsinki and Good Clinical Practice standards.

### Treatment

Fulvestrant (500 mg) was intramuscularly administered on day 1 of each cycle for a duration of 28 days (1 additional dosing on day 15 of cycle 1). Vinorelbine was taken orally at a dosage of 60 mg/m2 on days 1, 8, and 15, every 28 days. This combination was given until incidence of disease progression was observed, or intolerable AEs or withdrawal of consent by the patients occurred. Premenopausal patients were recommended to receive luteinizing hormone releasing hormone analog (LHRHa) treatment simultaneously. Additionally, if the patients were diagnosed with bone metastasis, bisphosphates were administered along with combination therapy.

During the study period, the imaging methods of either computed tomography or magnetic resonance imaging, which were used for evaluation at baseline, were recommended. Imaging evaluation was conducted every 8 weeks until disease progression occurred, as confirmed by the imaging examination. Initial efficacy evaluations of complete response (CR) or partial response (PR) were confirmed after at least 4 weeks.

### Outcomes

The primary endpoint was PFS, defined as the time from the beginning of treatment to progression or death from any cause. Disease progression was evaluated by investigators according to RECIST version 1.1. The secondary endpoints included overall survival (OS, defined as the period of time from the start of treatment to the occurrence of death from any cause), objective response rate (ORR, defined as the proportion of patients whose best response evaluation was CR or PR), disease control rate [DCR, the proportion of patients whose best response evaluation was CR or PR, and stable disease (SD)], and duration of response (DoR, defined as the period of time from the first confirmation of PR or CR to disease progression). Safety and tolerability assessments included AEs; serious AEs, as categorized and graded by the investigator according to National Cancer Institute Common Terminology Criteria for Adverse Events version 5.0; and the proportion of drug dosage adjustment because of AEs.

### Statistical analysis

The efficacy and safety in the full analysis set (defined as the total enrolled patients) were analyzed in R Software, version 3.2 (https://www.r-project.org/). PFS, OS, and DoR of the full analysis set were analyzed with the Kaplan–Meier method. The differences in either PFS or OS between the first-line and second-line subgroups were analyzed with the log-rank test and are presented as hazard ratios (HRs) with bilateral 95% confidence intervals (CIs). The Cox multiple regression model through univariate and multivariate analyses was used to further identify the potential independent predictors of PFS and baseline variables consisting of age (≤ 65 or > 65 years), ER level (≤ 50% or > 50%), Ki-67 level (≤ 30% or > 30%), PR level (≤ 10% or > 10%), pre- or postmenopausal status, number of metastatic lesions (≤ 2 or > 2), and metastatic status (such as visceral, liver, lung and bone metastasis). The secondary endpoints, either ORR or DCR, were based on the investigator-assessed confirmed best response. The number and proportion of responders or non-responders were analyzed by descriptive analysis with 95% CIs. The baseline demographics and safety consisting of the incidence of AE, serious AEs, and the proportion of dose re-adjustment due to AEs for the investigated drugs were descriptively analyzed. A *P* value of < 0.05 was considered statistically significant.

## Results

### Patient demographics and baseline characteristics

From January 26, 2018 to April 29, 2021, a total of 38 eligible patients were enrolled. Twenty-seven (71.1%) patients received the first-line treatment (defined as patients with recurrence and metastasis after adjuvant endocrine therapy for more than 1 year who did not receive treatment for recurrence and metastasis) and 11 (28.9%) patients received the second-line treatment (defined as patients with disease progression after first-line endocrine therapy or first-line chemotherapy). The baseline characteristics are presented in **Table 1**. Of the 11 patients receiving second-line treatment, 9 received first-line endocrine therapy, and the remaining 2 received first-line chemotherapy. The previous endocrine therapy consisted of aromatase inhibitors (AI) alone or in combination with everolimus or CDK4/6i ± LHRHa, whereas the chemotherapy regimens consisted of TP regimen (docetaxel + cisplatin) and GT (gemcitabine + docetaxel). Of the 30 patients receiving endocrine therapy in an adjuvant setting, 16 and 12 patients were administered tamoxifen and AI ± LHRHa, respectively. A total of 14 patients received AI therapy. The median age of the 38 enrolled patients was 55.5 years (range 39–72 years), and 76.3% of patients were ≤ 65 years of age. A total of 26 (68.4%) and 12 (31.6%) patients were premenopausal and postmenopausal, respectively. All patients were ER+, 31 patients (81.6%) had detected ER expression > 50%, and 6 patients (15.8%) had detected ER expression ≤ 50%. The number of patients with PR expression ≤ 10% and > 10% was 12 (31.6%) and 25 (65.8%), respectively.

**Table 1:** Baseline demographic characteristics (full analysis set)

Characteristics	All patients (*n* = 38)
Age, years	
Mean ± SD	55.7 (9.5)
Median (range)	55.5 (39–72)
≤ 65	29 (76.3%)
> 65	9 (23.7%)
Menopausal state	
Premenopausal	26 (68.4%)
Postmenopausal	12 (31.6%)
ER status	
ER ≤ 50%+	6 (15.8%)
ER > 50%+	31 (81.6%)
NE	1 (2.6%)
Ki-67 index	
Ki-67 ≤ 30%+	26 (68.4%)
Ki-67 > 30%+	10 (26.3%)
NE	2 (5.3%)
PR status	
PR ≤ 10%+	12 (31.6%)
PR > 10%+	25 (65.8%)
NE	1 (2.6%)
Site of recurrence and metastasis	
Viscera	30 (78.9%)
Liver	14 (36.8%)
Lung	22 (57.9%)
Bone	18 (47.4%)
Duodenum	1 (2.6%)
Lymph gland	14 (36.8%)
Breast/chest wall	6 (15.8%)
Number of metastatic sites	
≤ 2	25 (65.8%)
> 2	13 (34.2%)
Recurrence and metastasis	
Local recurrence	1 (2.6%)
Distant metastasis	37 (97.4%)
Number of treatment lines	
First-line treatment	27 (71.1%)
Second-line treatment	11 (28.9%)
Failure of first-line endocrine therapy	9 (23.7%)
AI ± LHRHa	5 (13.2%)
AI + CDK4/6i	2 (5.3%)
AI + everolimus ± LHRHa	2 (5.3%)
Failure of first-line chemotherapy	2 (5.3%)
Docetaxel + cisplatin	1 (2.6%)
Docetaxel + gemcitabine	1 (2.6%)
Endocrine therapy in adjuvant setting	
Yes	30 (78.9%)
TAM ± LHRHa	16 (42.1%)
AI ± LHRHa	12 (31.6%)
TAM followed by AI	1 (2.6%)
AI + everolimus	1 (2.6%)
No	8 (21.1%)

AI, aromatase inhibitors; CDK4/6i, cyclin-dependent kinase (CDK)4/6 inhibitors; ER, estrogen receptor; LHRHa, luteinizing hormone releasing hormone analog; NE, non-evaluable; PR, progesterone receptor; SD, standard deviation; TAM, tamoxifen.

A total of 26 patients (68.4%) had Ki-67 positivity ≤ 30%, and 10 (26.3%) patients had Ki-67 positivity > 30%. Most patients had visceral metastasis (30/38; 78.9%). Lung, liver, and bone metastasis were reported in 22/38 (57.9%), 14/38 (36.8%), and 18/38 (47.4%) patients, respectively. A total of 25/38 (65.8%) patients had ≤ 2 metastatic sites, and 13/38 (34.2%) patients had > 2 metastatic sites.

### Efficacy

**Figure 1:**
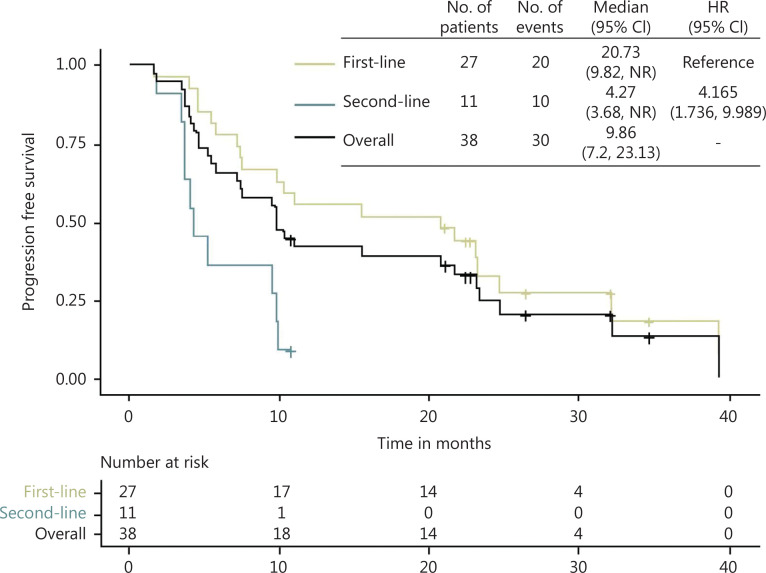
Kaplan–Meier curve for the PFS of the total population, first- and second-line subgroup. CI, confidence interval; HR, hazard ratio; NR, not reached.

**Figure 2:**
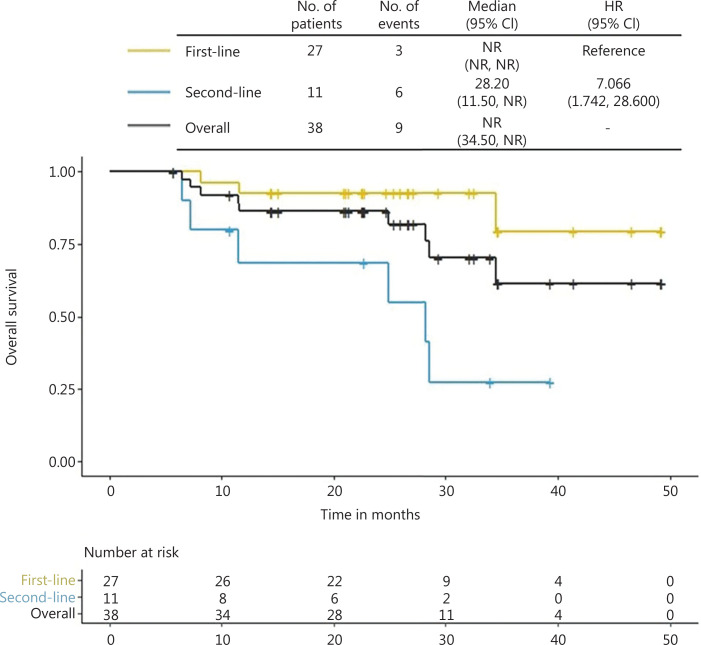
Kaplan–Meier curve for OS of the total population, first- and second-line subgroup. CI, confidence interval; HR, hazard ratio; NR, not reached.

Until October 20, 2021, the median follow-up time was 25.1 months (range 5.7–49.3 months), and all 38 patients were analyzed for efficacy outcomes. As shown in **Figure 1**, a total of 30 events occurred, comprising 20 first-line and 10 second-line events. The median PFS for the total population was 9.86 months (95% CI 7.2–23.13), and that for first-line treated patients was 20.73 months [95% CI 9.82 to not reached (NR)], which was significantly higher than that for second-line treated patients (4.27 months 95% CI 3.68 to NR). As shown in **Figure 2**, both the median OS for the total population and patients receiving first-line treatment was NR. The median OS for the patients receiving second-line treatment was 28.2 months (95% CI: 11.5 to NR).

**Table 2** summarizes the confirmed best overall response. The median DoR was 15.33 months (95% CI 7.23–22.54) for the total population, and 18.04 months (95% CI 15.05–22.93) and 5.75 months (95% CI 4.24–6.735) for the first- and second-line treated patients, respectively. Nonetheless no CR was achieved. A total of 15 patients achieved PR, including 12 patients receiving first-line treatment and 3 patients receiving second-line treatment. The ORR was 39.47% (15/38) for the total population, and the difference in ORR between patients receiving first-line (44.44%) and second-line (27.27%) treatment was more than 17%. Uncontrolled tumors (PD at first evaluation) were reported in 2 patients undergoing first-line treatment and 1 patient undergoing second-line treatment. The DCR was 92.11%, 92.59%, and 90.91% in the total population and in patients receiving first-line and second-line treatment, respectively.

**Table 2:** Confirmed optimum overall response

Response	Total population (*n* = 38)	First-line population (*n* = 27)	Second-line population (*n* = 11)
CR	0	0	0
PR	15	12	3
SD	20	13	7
PD	3	2	1
ORR (%)	39.47 (23.93, 55.01)	44.44 (25.70)	27.27 (0.95, 53.59)
DCR (%)	92.11 (83.54, 100.00)	92.59 (82.71, 102.47)	90.91 (73.92, 107.90)
Median DoR (months)	15.33 (7.23, 22.54)	18.04 (15.05, 22.93)	5.75 (4.24, 6.735)
Median PFS (months)	9.86 (7.20, 23.13)	20.73 (9.82, NR)	4.27 (3.68, NR)
Median OS (months)	NR	NR	28.20 (11.50, NR)

CR, complete response; DCR, disease control rate; DoR, duration of response; ORR, objective response rate; OS, overall survival; PD, progressive disease; PFS, progression free survival; PR, partial response; SD, stable disease.

**Figure 3:**
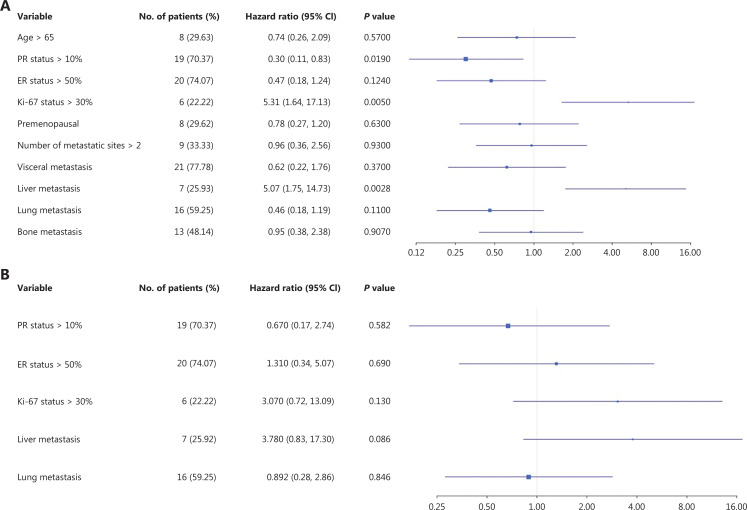
Forest plot for PFS in the first-line population: (A) univariate analysis and (B) multivariate analysis. CI, confidence interval; ER, estrogen receptor; PFS, progression free survival; PR, partial response.

The univariate and multivariate analyses are shown in **Figure 3A, 3B**. Univariate Cox proportional hazards regression analysis for PFS in the patients receiving first-line treatment demonstrated that PR > 10%+, Ki-67 > 30%, and liver metastasis were significantly correlated with PFS (*P* < 0.05, **Figure 3A**). Factors such as age, ER level, menopausal status, number of metastases, visceral metastasis, and lung or bone metastasis were not identified as independent factors associated with PFS. Further multivariate analysis (**Figure 3B**) indicated that patients with Ki-67 > 30% (HR = 3.07 95% CI 0.72–13.09, *P* = 0.13) and liver metastasis (HR = 3.78 95% CI 0.83–17.3, *P* = 0.086) had poorer PFS.

**Figure 4:**
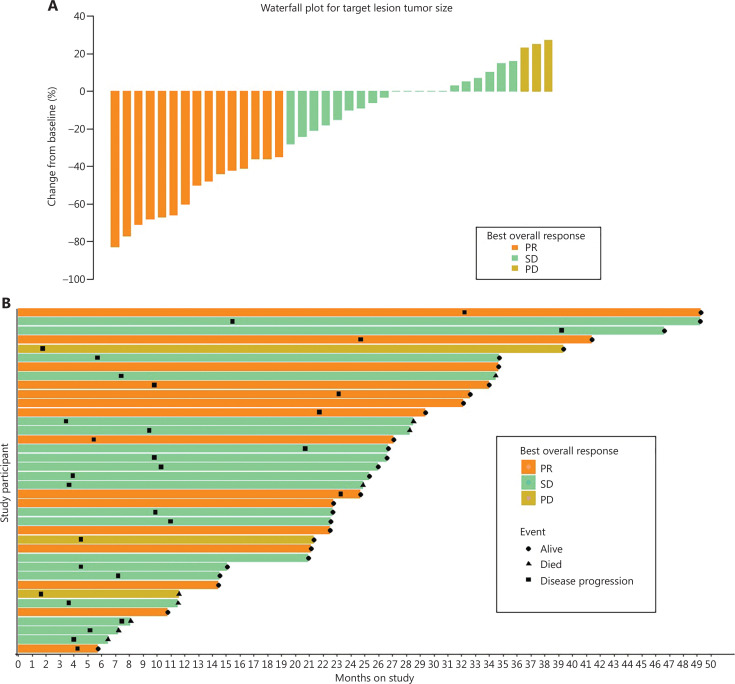
Tumor change and the best response during the follow-ups. (A) Waterfall plot of tumor changes in target lesion size. (B) Swimmer plot of the optimum response (PR/SD/PD) of each patient during the corresponding follow-up time (month). PR, partial response; PD, progressive disease; SD, stable disease.

The tumor changes in target lesions and the best response during follow-up are shown in **Figure 4A, 4B** as Waterfall and Swimmer plots, respectively.

### Safety and tolerability

The median treatment time for the fulvestrant plus vinorelbine regimen was 9.2 months (1.8–35.9 months). Eight patients (21.1%) underwent vinorelbine dose adjustment because of AEs, including 7 dose reductions and 1 withdrawal. Among these, only 1 patient underwent dose adjustment more than once. The most frequent AEs leading to vinorelbine dose adjustment were nausea or vomiting (7/8, 87.5%), and no dose adjustment was required for fulvestrant. Ondansetron tablets were most commonly used to alleviate the symptoms of nausea and vomiting. As summarized in **Table 3**, the most common AEs of any grade were transaminase elevation (44.74%), nausea (36.84%), vomiting (15.79%), diarrhea (15.79%), leucopenia (13.16%), and neutropenia (10.53%). The AEs were mostly of grade 1 or 2 in severity. Only 2 AEs (vomiting and fatigue) were of grade 3, and no grade 4–5 AEs were observed.

**Table 3:** Treatment-emergent adverse events

AE, *n* (%)	Treatment regimens: fulvestrant (500 mg) plus oral vinorelbine (60 mg/m^2^), *n* = 38				
	All grades	Grade 1	Grade 2	Grade 3	Grade 4 or 5
Any	33 (86.84)	29 (76.32)	8 (21.05)	2 (5.26)	0
Nausea	14 (36.84)	13 (34.21)	1 (2.63)	0	0
Vomiting	6 (15.79)	4 (10.53)	1 (2.63)	1 (2.63)	0
Diarrhea	6 (15.79)	5 (13.16)	1 (2.63)	0	0
Decreased appetite	1 (2.63)	1 (2.63)	0	0	0
Alopecia	3 (7.89)	3 (7.89)	0	0	0
Fatigue	1 (2.63)	0	0	1 (2.63)	0
Fever	2 (5.26)	2 (5.26)	0	0	0
Transaminase elevation	17 (44.74)	15 (39.47)	2 (5.26)	0	0
Neutropenia	4 (10.53)	1 (2.63)	3 (7.89)	0	0
Leucopenia	5 (13.16)	3 (7.89)	2 (5.26)	0	0
Anemia	1 (2.63)	1 (2.63)	0	0	0
Thrombocytopenia	1 (2.63)	0	1 (2.63)	0	0

AE, adverse event.

## Discussion

Although combined chemo-endocrine therapy has been reported to have advantages as an adjuvant therapy for breast cancer^7^, it remains an exploratory treatment for advanced stages of breast cancer. To our knowledge, this study is the first clinical trial to investigate the efficacy and safety of fulvestrant in combination with oral vinorelbine for the treatment of advanced HR+/HER2− breast cancer, among studies worldwide.

In the FALCON study, a PFS of 16.6 months was reported with fulvestrant monotherapy in the treatment of naive postmenopausal advanced or metastatic breast cancer^1^, whereas another study on fulvestrant plus anastrozole, or vinorelbine monotherapy as the first-line treatment has reported a PFS of 15 months or 7.7 months, respectively^8,9^. The results of our study indicated that the median PFS in the patients receiving first-line treatment who experienced recurrence and metastasis after adjuvant endocrine therapy was 20.73 months, thus indicating an absolute benefit, although comparison among studies must be undertaken with great caution.

Endocrine drugs are effective primarily on HR+ tumor cells, whereas HR− tumor cells are more sensitive to chemotherapeutic agents. Because of tumor heterogeneity, HR+ breast cancer contains HR− tumor cells. Thus, combination chemo-endocrine therapy is expected to increase the efficacy of endocrine therapy for HR+ breast cancer. Moreover, most patients with HR+ breast cancer are likely to develop endocrine resistance. The mechanisms underlying endocrine resistance are highly complicated, involving mutations affecting various genes (such as ER, aromatase, and tyrosine kinase receptor) and signaling pathways (such as phosphatidylinositol 3-kinase and mitogen-activated protein kinase), variations in transcription factors (such as *MYC*, *FOXA1*, *CTCF*, or *TBX3*), epigenetic changes, metabolic reprogramming of tumor cells, or tumor microenvironment changes^10^. Individualized regimens should be considered according to the specific mechanisms of resistance. However, identifying the mechanisms of drug resistance encountered in clinical practice is challenging. Therefore, combining endocrine therapy with a chemotherapy agent of broad-spectrum effect is favorable.

Although chemotherapy may be effective against endocrine resistant tumors, multidrug resistance (MDR) in chemotherapy remains a major concern. Vinorelbine, a commonly used chemotherapeutic regimen in breast cancer, can cause MDR mainly through a permeability glycoprotein (P-gp) mediated mechanism of drug resistance^11^. Despite MDR, previous studies have shown that a combination of biweekly vinorelbine with docetaxel yields a median survival of 19.6 months with well-tolerated toxicity^12^. Furthermore, vinorelbine has demonstrated similar clinical benefits in combination with lapatinib and vinorelbine in patients whose cancer progressed on both trastuzumab and lapatinib treatments^13^. In contrast, experimental studies have shown that fulvestrant inhibits P-gp function and reverses P-gp mediated drug resistance in MDR cell lines, such as Bads-200 and Bats-72^14^. In addition, *in vivo* and *in vitro* experiments have indicated that fulvestrant downregulates the levels of chemotherapy resistance factors (such as Bcl2, MRP1, and MAPT) in MCF-7 and ZR75-1 cell lines, and increases the sensitivity of chemotherapy agents^6^. Therefore, fulvestrant may reverse the drug resistance caused by vinorelbine, and the combination of these agents may be advantageous through providing synergistic anti-tumor effects. Regarding this possibility, a study has reported fulvestrant might be a novel strategy to reverse ER-mediated chemoresistance or sensitize ER+ breast tumors to vinca alkaloids and possibly other chemotherapeutic agents^15^.

Our study showed that the median PFS and DoR in patients receiving first-line treatment were longer than 18 months. The trend of improvement in OS was consistent with PFS, and the ORR was 44.44%. Therefore, the combination regimen in the first-line treatment of HR+/HER2− advanced breast cancer conferred clear advantages in both rapid tumor control and prolonging overall survival. In addition, the DCR for the second-line treatment exceeded 90%, thus suggesting that the regimen also had promising effectiveness on short-term tumor control after the first-line treatment. Univariate and multivariate analyses showed that patients with Ki-67 ≤ 30% without liver metastasis might be the preferred group for first-line treatment of this regimen. Moreover, the results also showed that patients with visceral metastases or > 2 metastatic sites could still achieve the same efficacy as patients without visceral metastases or ≤ 2 metastases. Overall, combined chemo-endocrine therapy is expected to be a feasible option for HR+ HER2− terminal-stage breast cancer.

Safety analysis revealed that most AEs were of grade 1/2 (76.32% and 21.05%, respectively). Only 2 patients had AEs of grade 3, and none had grade 4/5 AEs. The incidence of decreased leucopenia, neutropenia, and diarrhea was 13.16%, 10.53%, and 15.79%, respectively, and above AEs were of grade 1/2, which were substantially lower than those previously reported in CDK4/6i plus endocrine therapy^3^. Eight patients (21.05%) underwent dose reduction for vinorelbine, and only 1 patient (2.63%) discontinued the medication because of AEs. Thus, the treatment with fulvestrant combined with oral vinorelbine was well tolerated and may be an option for patients with CDK4/6i intolerance.

At the onset of this trial, CDK4/6i were not easily accessible and affordable in China, although this availability has changed in recent years. Nevertheless, some patients cannot tolerate the toxicity of CDK4/6i, such as hematological effects or diarrhea. Thus, this novel combination may be a first-line treatment choice for some patients. Furthermore, for patients showing cancer progression on CDK4/6i combined with AI, fulvestrant plus vinorelbine may also be a second-line option.

## Conclusions

In conclusion, the regimen with fulvestrant in combination with oral vinorelbine was efficacious, tolerable, and convenient to use in patients with HR+/HER2− advanced breast cancer and hence may be considered in clinical practice. However, this study has several limitations. Because this was an exploratory single-arm study, the sample size was limited, and the enrolled patients had previous treatment history. Therefore, to provide further supporting evidence, randomized controlled trials are needed with larger sample sizes in this specific population.
